# The integrative role of melatonin in psychiatric disorders: A systematic review of evidence from circadian biology, lifestyle medicine, and psychoneuroimmunology

**DOI:** 10.37796/2211-8039.1682

**Published:** 2025-12-01

**Authors:** Ho Bao Chau Le, Kollawat Somsri, Thansita Bhunyakarnjanarat, Nattiya Hirankarn, Asada Leelahavanichkul

**Affiliations:** a Chulalongkorn University International Medical Sciences Program, Faculty of Medicine, Chulalongkorn University, Bangkok, Thailand; b Department of Microbiology, Faculty of Medicine, Chulalongkorn University, Bangkok, Thailand; c Center of Excellence in Immunology and Immune-mediated Diseases, Chulalongkorn University, Bangkok, Thailand; d Center of Excellence in Translational Research in Inflammation and Immunology (CETRII), Chulalongkorn University, Bangkok, Thailand; e Chulalongkorn University International Medical Program (CU-MEDi), Faculty of Medicine, Chulalongkorn University, Bangkok, Thailand

**Keywords:** Circadian rhythm, Lifestyle medicine, Melatonin, Psychiatric disorders, Psychoneuroimmunology

## Abstract

Melatonin, a hormone produced by the pineal gland, is known for regulating circadian rhythms and has emerging therapeutic potential in psychiatric disorders. This systematic review examines evidence from clinical and preclinical studies to assess melatonin's efficacy in the following conditions: depression, anxiety, bipolar disorder, and schizophrenia. Beyond its role in circadian regulation, melatonin exhibits antioxidative, anti-inflammatory, and immunomodulatory properties, which intersect with nutrition, lifestyle medicine, and psychoneuroimmunology (PNI).

The review finds that melatonin improves sleep quality, restores circadian balance, and modulates stress-related neuroimmune pathways. It also supports neuroplasticity and reduces oxidative stress, contributing to resilience against psychosocial and environmental stressors. Lifestyle factors, including diet, exercise, and sleep hygiene, enhance melatonin's effects, positioning it as a valuable component of multimodal treatment strategies. Within the PNI framework, melatonin facilitates communication between the nervous and immune systems, offering potential for targeted psychiatric interventions.

This synthesis underscores melatonin's promise as a therapeutic and adjunctive strategy in personalized mental health care. Future research should prioritize rigorous clinical trials, biomarker-driven patient stratification, and integrative approaches combining melatonin with lifestyle interventions and digital health tools to optimize its therapeutic impact.

## 1. Introduction

Psychiatric disorders represent a growing global health burden, contributing substantially to disability and reduced quality of life across populations [1]. Despite advances in pharmacological and psychotherapeutic interventions, many patients experience partial response or relapse, underscoring the need for more integrative and individualized therapeutic strategies.

Converging evidence has highlighted the interplay between circadian rhythms, nutrition, lifestyle medicine, and psychoneuroimmunology (PNI) as crucial determinants of mental health. Circadian disruption has been linked with dysregulated immune responses and altered neurochemical balance, contributing to the pathophysiology of depression, bipolar disorder, and schizophrenia [2]. Nutritional choices and lifestyle modifications, namely adherence to anti-inflammatory diets, regular physical activity, adequate sleep hygiene, and time-restricted eating, have shown promise in mitigating neuroinflammation and improving mental well-being [3]. Similarly, PNI has illuminated the complex bidirectional interactions between the brain, immune, and endocrine systems that underpin psychiatric disorders [4].

Within this framework, melatonin emerges as a compelling candidate that bridges these domains. Secreted by the pineal gland under circadian regulation, melatonin exerts multifaceted effects on sleep–wake cycles, immune modulation, oxidative stress, and neuroendocrine signaling [5]. Its capacity to integrate circadian, nutritional, lifestyle, and neuroimmune influences positions it as a unique therapeutic target in psychiatry.

This review synthesizes current evidence on the integrative role of melatonin in mental health and evaluates its potential as an adjunctive therapy for psychiatric disorders. It also provides insights to guide future interdisciplinary research and clinical applications.

## 2. Literature search methodology

This review followed systematic search and screening methods, and reporting was informed by PRISMA 2020 guidelines where applicable, with a focus on systematic search and screening methods followed by narrative synthesis. A detailed protocol was prospectively registered on the Open Science Framework. A comprehensive search of PubMed, Embase, CENTRAL, Web of Science, Scopus, and PsycINFO was carried out from inception to July 2025, using the following key terms: melatonin, circadian rhythm, psychiatric disorders, depression, anxiety, bipolar disorder, schizophrenia, psychoneuroimmunology, and related synonyms (combined with controlled vocabulary and free-text terms). Eligible studies included human trials and observational research on melatonin or its analogs in psychiatric populations; animal-only studies, case reports, conference abstracts, and duplicates were excluded. Risk of bias was assessed using appropriate tools for each study type. Outcomes included sleep quality, circadian regulation, neuropsychiatric symptom severity, neuroimmune markers, and tolerability.

**Fig. 1:**
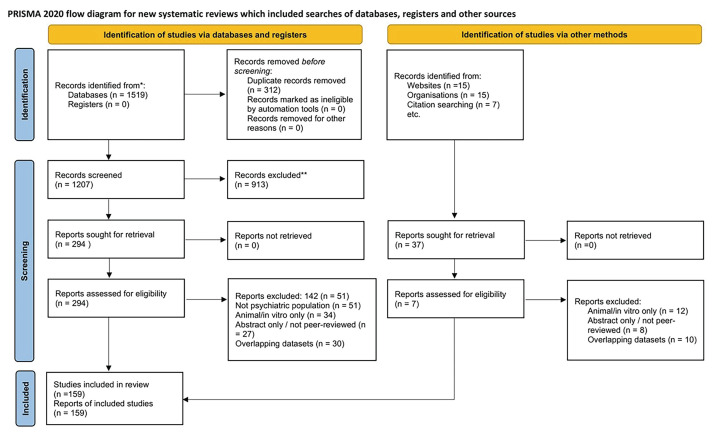
The study selection process is summarized in the PRISMA 2020 flow diagram. It illustrates the number of records identified, screened, excluded, and ultimately included in the review, together with reasons for exclusion at each stage.

Data were synthesized narratively due to study heterogeneity, precluding quantitative meta-analysis, though random-effects meta-analysis was considered. Animal and in vitro studies were excluded, but key preclinical findings were included to contextualize mechanistic plausibility. Two reviewers independently screened titles, abstracts, and full texts, resolving discrepancies by consensus. The selection process is summarized in a PRISMA 2020 flow diagram (Fig. 1). Of 1519 records retrieved, 159 studies met eligibility criteria after duplicate removal and screening.

## 3. Melatonin and circadian biology

Melatonin is an indoleamine primarily synthesized from tryptophan in the pineal gland through a multi-step enzymatic pathway. Its secretion follows a robust circadian pattern, with peak production during the dark phase and near-complete suppression by light exposure to the retina [6,7]. This rhythmic secretion is under the control of the suprachiasmatic nucleus (SCN) of the hypothalamus, the central circadian pacemaker, which conveys photic signals through a multisynaptic pathway to regulate pineal activity [8]. As a circadian synchronizer, melatonin exerts feedback onto the SCN to stabilize circadian phase and promote entrainment of peripheral clocks [9]. This reciprocal regulation ensures coherence between central and peripheral oscillators, thereby coordinating sleep–wake cycles, thermoregulation, endocrine rhythms such as cortisol, growth hormone, and neurochemical balance across brain regions [10].

Disruptions in circadian rhythms are frequently reported with psychiatric disorders. Reduced or phase-shifted melatonin secretion has been observed in patients with major depressive disorder, bipolar disorder, and schizophrenia [11,12]. These alterations are linked with sleep disturbances, impaired hormonal homeostasis, and neuroimmune dysregulation, all of which exacerbate psychiatric symptoms.

Notably, exogenous melatonin and its analogs have shown efficacy in correcting circadian misalignment and improving sleep quality, suggesting a potential therapeutic pathway in psychiatry [13]. Together, these findings highlight melatonin's central role as both an output and regulator of circadian rhythms, with implications for psychiatric outcomes when its synthesis or signaling is disrupted.

## 4. Melatonin and psychoneuroimmunology (PNI)

Psychoneuroimmunology (PNI) explores the bidirectional interactions between the nervous, immune, and endocrine systems, providing a framework for understanding how stress and immune dysregulation contribute to psychiatric disorders [4,14]. Within this context, melatonin emerges as a molecule capable of modulating neuroimmune processes through its antioxidant, anti-inflammatory, and endocrine-regulating properties.

Melatonin exerts potent anti-inflammatory and immunomodulatory effects by inhibiting proinflammatory cytokines (TNF-α, IL-6, IL-1β) while enhancing anti-inflammatory mediators (IL10) [15]. It also regulates innate and adaptive immune responses by modulating T-helper cell balance, promoting regulatory T-cell activity, and influencing microglial polarization [5]. In parallel, melatonin reduces oxidative stress by directly scavenging free radicals and upregulating antioxidant enzymes, thereby protecting neural tissue from inflammatory damage [16].

Disruptions in the hypothalamic–pituitary–adrenal (HPA) axis are central to stress-related psychiatric disorders. Melatonin has been shown to counteract hyperactivation of the HPA axis, restoring glucocorticoid sensitivity and attenuating stress-induced neuroinflammation [17]. These mechanisms suggest that melatonin functions as a key mediator linking circadian and immune regulation with psychiatric outcomes.

Preclinical evidence supports melatonin's role in mitigating neuroimmune dysfunction relevant to psychiatry. In animal models, melatonin administration reduces microglial activation, suppresses neuroinflammatory cascades, and prevents stress-induced behavioral abnormalities [18]. Clinically, reduced melatonin levels have been associated with elevated inflammatory markers and symptom severity in patients with depression and schizophrenia [19]. Some clinical studies have reported that the use of exogenous melatonin or its agonists (e.g., agomelatine, ramelteon) demonstrate improvements in sleep, mood regulation, and inflammatory biomarkers, further supporting its therapeutic potential [20].

Collectively, these findings position melatonin as a crucial interface in PNI, capable of attenuating neuroinflammation, oxidative stress, and HPA-axis dysregulation, thereby offering mechanistic insights into its role in psychiatric disorders.

## 5. Melatonin, nutrition, and lifestyle medicine

Melatonin production is not only endogenously regulated by the pineal gland but can also be influenced by nutritional and lifestyle factors. Dietary sources of melatonin include a variety of plant-based foods including cherries, walnuts, rice, and grapes, as well as animal-derived products like milk and eggs [21]. In addition, tryptophan-rich diets support melatonin biosynthesis by providing a key precursor for serotonin and subsequent melatonin production [22]. Dietary patterns such as the Mediterranean diet, characterized by high intake of fruits, vegetables, legumes, and whole grains, have been associated with improved sleep quality, reduced inflammation, and higher circulating melatonin levels [23]. Time-restricted eating and intermittent fasting also appear to enhance circadian alignment and melatonin secretion, potentially conferring resilience against mood and sleep disorders [24].

Lifestyle behaviors exert a parallel influence on melatonin rhythms. Good sleep hygiene, including consistent bedtimes and avoidance of artificial light exposure at night, supports nocturnal melatonin secretion and circadian synchronization [25]. Exposure to natural light during the day and reduced evening screen use are particularly important for entraining circadian rhythms and sustaining melatonin amplitude. Physical activity has also been shown to modulate melatonin levels and improve circadian stability, with regular exercise linked with improved sleep quality and lower risk of depression [26]. Daily routines that reinforce consistent timing of sleep, meals, and activity further strengthen circadian signals and melatonin regulation.

Melatonin acts as a mediator of these nutritional and lifestyle effects on mental health. By integrating signals from diet, light, and behavior, melatonin helps restore circadian homeostasis and modulate neuroimmune and neuroendocrine pathways relevant to psychiatric disorders. For example, lifestyle modifications that enhance melatonin rhythms have been associated with reduced neuroinflammation, improved mood regulation, and better cognitive performance [27].

This evidence supports melatonin as both a biological marker and a mechanistic mediator linking nutrition and lifestyle interventions with circadian health and psychiatric well-being. As illustrated in Table 1, a wide range of dietary and lifestyle variables modulate melatonin rhythms through distinct neuroendocrine and immune pathways, with downstream effects on psychiatric health [28,40]. From our perspective, the integration of melatonin supplementation with lifestyle-based interventions (e.g., time-restricted feeding, reduced evening blue light, structured exercise) holds considerable promise for synergistic effects. However, multi-domain randomized trials remain lacking, thus representing a critical gap.

## 6. Clinical implications of melatonin for psychiatric disorders

Melatonin upholds multifaceted roles in regulating circadian rhythms, balancing oxidative stress, and modulating immune responses. These properties position it as a promising candidate for psychiatric research. Growing evidence indicates that disruptions in circadian rhythms, heightened oxidative stress, and immune dysregulation are central to the pathophysiology of major psychiatric disorders. Against this backdrop, melatonin and its analogs have been investigated as both adjunctive and primary therapeutic options, with results that are encouraging but inconsistent.

Melatonin's therapeutic potential lies in its ability to restore sleep–wake cycles, regulate neurotransmitter activity, and reduce oxidative and inflammatory processes. Its clinical applications have been explored across various psychiatric conditions, yielding benefits that vary by disorder and context. The clinical applications of melatonin across different psychiatric conditions are synthesized in Table 2, providing a concise overview of its benefits, underlying mechanisms, and current limitations.

These findings underscore the potential for melatonin as a safe adjunctive therapy, particularly for disorders involving circadian or sleep disturbances. However, the evidence base is limited by methodological inconsistencies, including small sample sizes, short study durations, and a focus on secondary outcomes like sleep quality rather than primary psychiatric symptoms. Variations in melatonin formulations—immediate-release, prolonged-release, or synthetic analogs like agomelatine—further complicate interpretation and hinder the establishment of standardized dosing guidelines. The efficacy of melatonin also varies across populations. It is most effective in conditions marked by sleep or circadian dysfunction, including ASD or insomnia in MDD, but its impact on core psychiatric symptoms, like delusions in schizophrenia or mood instability in bipolar disorder, remains limited. Additionally, the lack of long-term studies leaves questions about sustained benefits and potential tolerance unresolved.

Despite these limitations, melatonin possesses a favorable safety profile, with low risk of dependence and mild side effects, making it an attractive alternative to pharmacotherapies with greater toxicity. Beyond its role in sleep regulation, the diverse effects of melatonin—properties involving antioxidation, anti-inflammation, and neuroprotectivity—suggest broader therapeutic potential. However, these mechanisms require further rigorous clinical validation to confirm their relevance in psychiatric treatment.

When viewed holistically, clinical evidence suggests that melatonin is most robust in stabilizing sleep and circadian rhythms, while evidence for direct effects on core psychiatric symptoms remains weak. Based on this synthesis, we recommend viewing melatonin primarily as an adjunctive tool, particularly in patients with comorbid sleep disruption.

### 6.1. Comparative perspectives: Melatonin versus established interventions

While melatonin and its analogs demonstrate encouraging effects in psychiatric care, it is critical to evaluate their role relative to existing therapeutic standards. Compared to cognitive–behavioral therapy for insomnia (CBT-I), which remains the gold-standard non-pharmacological intervention, melatonin provides a more rapid improvement in sleep onset but lacks the durable efficacy and relapse prevention observed with CBT-I [13]. In contrast to hypnotic and sedative agents such as benzodiazepines or Z-drugs, melatonin offers a superior safety profile, with negligible risk of dependence, cognitive impairment, or falls—adverse effects that are particularly problematic in older adults. However, hypnotics often yield stronger short-term efficacy in severe insomnia, underscoring the comparatively modest clinical effect size related to melatonin.

Relative to antidepressants and SSRIs, melatonin and agomelatine provide dual chronobiotic (a regulator of biological rhythms) and mood-regulating actions, with better tolerability and fewer metabolic or sexual side effects [42]. Nevertheless, SSRIs and SNRIs remain more effective in addressing the core affective and cognitive symptoms of depression, whereas the benefits of melatonin appear largely confined to circadian realignment and sleep quality [41]. Similarly, in schizophrenia, melatonin supplementation improves sleep and mitigates antipsychotic-induced metabolic complications, but it exerts little impact on primary psychotic manifestations [19,48]. Taken together, melatonin occupies a unique therapeutic niche: it is safe, circadian-targeted, and well-suited for adjunctive use, yet unlikely to supplant first-line pharmacological or psychotherapeutic approaches. Overall, we argue that melatonin should be regarded primarily as a circadian and sleep-regulating agent rather than direct treatment for psychiatric disorders. Nevertheless, when embedded within multimodal strategies (pharmacotherapy, CBT, and lifestyle modification), melatonin could serve as a valuable cornerstone in the shift toward integrative and precision psychiatry.

## 7. Emerging perspectives and future directions

The evolving field of melatonin research highlights the need for integrative and precision-oriented approaches. Beyond traditional clinical trials, multi-omics technologies now allow for deeper insights into the biological underpinnings of effects induced by melatonin. Genomic and epigenomic profiling can identify polymorphisms in MTNR1A/B and clock genes that modulate individual responses [54], while metabolomic and transcriptomic studies provide data on downstream pathways involving inflammation, oxidative stress, and neuroplasticity [19]. Such approaches may enable the development of biomarker-based stratification, ensuring that melatonin is targeted to patient subgroups most likely to benefit.

Simultaneously, artificial intelligence (AI) and machine learning are emerging as powerful tools for data integration. By combining genetic, clinical, and behavioral data, predictive algorithms may help identify optimal dosing strategies and anticipate adverse interactions in polypharmacy contexts. AI-driven precision psychiatry could thus move melatonin from a “one-size-fits-all” supplement toward a tailored therapeutic option.

In parallel, digital health and wearable technologies offer new opportunities for real-world monitoring. Actigraphy and wrist-worn sensors have demonstrated reliability in detecting circadian phase shifts and melatonin-related rhythm alterations in psychiatric populations [55,56]. Recent validation studies confirm that consumer-grade devices, including smartwatches and mobile applications, approximate polysomnography with acceptable accuracy in both clinical and community samples [57,58]. These tools enable ecological, longitudinal assessment of sleep–wake cycles, light exposure, and lifestyle factors, creating feedback loops where melatonin interventions can be dynamically adjusted. Table 3 outlines key limitations identified in the current literature, along with proposed strategies and tools to bridge these gaps and foster more precise and effective applications in mental health care.

Taken together, the integration of omics profiling, AI analytics, and wearable monitoring represents a promising frontier for precision chronotherapy. This multi-dimensional framework positions melatonin not merely as a circadian modulator but as a candidate molecule at the crossroads of biology, technology, and psychiatry, capable of bridging basic science with individualized clinical care.

**Fig. 2:**
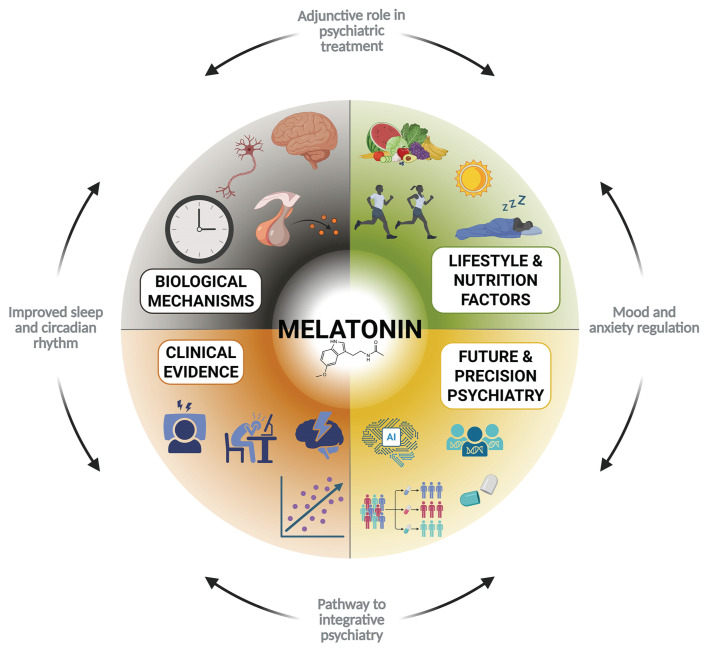
Integrative role of melatonin in psychiatry. Melatonin acts as a central hub linking biological mechanisms, lifestyle and nutritional factors, and clinical evidence, providing a foundation for therapeutic applications in psychiatric disorders and guiding future directions for precision psychiatry. Top left: Biological Mechanisms – circadian rhythm regulation, antioxidative and anti-inflammatory effects, neuroplasticity, HPA axis modulation; Top right: Lifestyle and Nutrition Factors – diet, light exposure, sleep quality and shift work, physical activity and stress management; Bottom left: Clinical Evidence – sleep stabilization, adjunctive therapy for mood, relapse prevention, trials and results; Bottom right: Future and Precision Psychiatry – dosing optimization, patient stratification, genetic and epigenetic moderators, AI-driven personalization.

This review has certain limitations. The included studies were highly heterogeneous in terms of dosing strategies, formulations, populations, and outcome measures, which limit comparability and the strength of generalizable conclusions. Moreover, because we used a narrative rather than a meta-analytic approach, we were able to integrate mechanistic, clinical, and lifestyle perspectives but could not provide pooled effect sizes or formally assess publication bias. To summarize the multifaceted role of melatonin in psychiatry, we present an integrative framework (Fig. 2). This model highlights how melatonin connects biological mechanisms, lifestyle and nutritional factors, clinical evidence, and future directions in precision psychiatry, providing a unifying perspective for its therapeutic potential.

## 8. Conclusion

Melatonin extends beyond its role as a sleep-regulating hormone, emerging as a pleiotropic molecule that intersects with circadian regulation, immune regulation, lifestyle factors, and psychiatric outcomes. This review highlights its therapeutic potential across a range of psychiatric disorders, including mood, anxiety, and neurodevelopmental conditions, particularly when circadian disruption or immune dysregulation is prominent. Randomized controlled trials and meta-analyses support melatonin's ability to improve sleep and provide adjunctive mood stabilization, yet inconsistencies in study design, dosing, and outcome measures hinder clear clinical recommendations.

Future research should leverage multi-omics profiling, AI-driven analytics, and digital health tools to identify optimal responders and tailor interventions to individual chronotypes and biological profiles. Such advancements could elevate melatonin from a widely used supplement to a cornerstone of precision chronotherapy in psychiatry.

Ultimately, melatonin serves as a bridge between molecular biology, lifestyle, and mental health. By integrating mechanistic insights with cutting-edge technologies, it holds promise not only for enhancing psychiatric treatment but also for shaping a translational framework for personalized, integrative medicine in the modern era.

## Figures and Tables

**Table 1:** Nutritional and lifestyle factors affecting melatonin and psychiatric health.

Factor	Effect on Melatonin	Key Mechanisms	Psychiatric Implications
Dietary nutrients (tryptophan, Bvitamins, Mg, folate)	Enhance synthesis of serotonin → melatonin	Precursor and cofactor supply for AANAT/HIOMT enzymes	e.g., Deficiencies linked with depression, poor sleep, cognitive decline [28]
Pro-/antiinflammatory diet	Western diets may suppress melatonin; Mediterranean/plant-based support it	Cytokine-driven IDO activation diverts tryptophan to kynurenine; antioxidants/polyphenols protect pineal	Inflammation-associated depression, bipolar disorder relapses; improved outcomes with anti-inflammatory diets [29]
Meal timing (chrononutrition)	Late-night eating delays/blunts nocturnal melatonin surge	Desynchronization of SCN and peripheral clocks	Irregular meals associated with depression, bipolar instability; time-restricted feeding improves mood [30]
Sleep quality & shift work	Poor/fragmented sleep and night shifts lower amplitude or invert melatonin	Cortisol suppression, cytokine elevation, SCN misalignment	Insomnia precipitates depression/anxiety; circadian disruption worsens bipolar and schizophrenia symptoms [31,32]
Light exposure	Evening blue light suppresses melatonin; morning sunlight enhances rhythm	Retinal SCN input inhibits pineal AANAT; altered receptor sensitivity	Linked with depression, SAD, mania induction; light/dark therapy stabilizes mood [33]
Physical activity	Regular exercise increases nocturnal melatonin and advances onset	Thermoregulation, SCN entrainment, anti-inflammatory adaptation	Exercise reduces depression/anxiety, stabilizes bipolar rhythms [34,35]
Stress & HPA activity	Chronic stress/cortisol suppress melatonin; relaxation restores	Cortisol–melatonin antagonism; cytokine-mediated IDO activation	Stress-induced depression, PTSD insomnia; melatonin agonists improve resilience [36,37]
Substance use (caffeine, nicotine, alcohol)	Evening use suppresses or delays melatonin	Adenosine receptor antagonism, catecholamine release, CYP metabolism competition	Exacerbates insomnia, anxiety, and relapses risk; abstinence improves mood stability [38–40]

**Table 2:** Clinical evidence of melatonin use in psychiatric disorders.

Disorder	Population/Study type	Formulation (Dose)	Benefits	Mechanisms	Limitations
MDD [10,41,42]	Adults, RCTs & adjunctive trials	Agomelatine; melatonin (2–5 mg)	Sleep improvement, mood regulation	Circadian realignment, BDNF, antioxidative	Small sample sizes, mixed results on core depression.
Bipolar Disorder [43–45]	Adults, pilot RCTs	Melatonin 3–5 mg	Sleep stabilization, relapse reduction	Chronobiotic effect, clock gene modulation	Limited trials, no standardized dose.
GAD [20,46]	Adults, perioperative & small RCTs	3–6 mg	Reduced anxiety, as sedation substitute	GABAergic modulation	Not validated for chronic GAD
Schizophrenia [19,47–49]	Adults on antipsychotics	2–5 mg	Better sleep, fewer metabolic side effects	Antioxidative, circadian alignment	Little effect on psychosis symptoms
ASD [50,51]	Children & adolescents	Prolongedrelease 2–6 mg	Sleep onset, daytime behavior	Circadian correction, melatonin synthesis deficits	Genetic variability (CYP1A2), relapses after withdrawal
ADHD [52,53]	Pediatric RCTs	Prolongedrelease 2–5 mg	Sleep onset, better attention	Sleep regulation, circadian rhythm	Not addressing hyperactivity core symptoms

**Table 3:** Research gaps and future directions in melatonin psychiatry.

Area	Current limitations	Research needs	Potential tools/Approaches
Dosing strategies	Inconsistent doses (0.5–10 mg) across trials; varied formulations	Standardized RCTs comparing immediate vs. prolonged release	Dose–response studies, pharmacokinetics
Patient heterogeneity	Mixed outcomes due to diverse populations	Stratification by age, sex, comorbidities	Biomarker-driven subgroup analyses
Genetic/epigenetic factors	Limited data on MTNR1A/B polymorphisms, clock gene variants	Identify moderators of melatonin response	Genomic/epigenomic profiling
Long-term efficacy/safety	Few longitudinal studies	Multi-year RCTs, safety monitoring in psychiatry	Cohort studies, registries
Drug–drug interactions	Poorly studied in polypharmacy settings	Evaluation with psychotropics, anticoagulants	Pharmacovigilance, in vitro metabolism studies
Integration with lifestyle & nutrition	Evidence fragmented across domains	Multi-domain intervention trials	Multidisciplinary, integrative study designs
Precision psychiatry	Lack of predictive models	AI-based personalization of melatonin therapy	Machine learning, multiomics integration

## Abbreviations

ADHD: Attention deficit hyperactivity disorder
ASD: Autism spectrum disorder
CBT-I: Cognitive behavioral therapy for insomnia
GAD: Generalized anxiety disorder
HPA: Hypothalamic–pituitary–adrenal
MDD: Major depressive disorder
PNI: Psychoneuroimmunology
SCN: Suprachiasmatic nucleus
SNRI: Serotonin–norepinephrine reuptake inhibitor
SSRI: Selective serotonin reuptake inhibitor
AI: Artificial intelligence
